# Dual‐Targeted Therapy in Cardiometabolic Risk: A Meta‐Analysis of Telmisartan‐Based Combinations for Hypertension and Dyslipidemia

**DOI:** 10.1002/clc.70211

**Published:** 2025-11-26

**Authors:** Rabia Asim, Tazheen Saleh Muhammad, Saad Ahmed, Laiba Khurram, Bazil Azeem, Mata‐e‐Alla Doggar, Abdullah Naveed Muhammad, Rahul Chikatimalla, Sowjanya Kapaganti, Himaja Dutt Chigurupati, Binish Qureshi, Harigopal Sandhyavenu, Sivaram Neppala

**Affiliations:** ^1^ Department of Cardiology Shaheed Mohtarma Benazir Bhutto Lyari Medical College Karachi Pakistan; ^2^ Department of Cardiology, Dow Medical College Dow University of Health Sciences Karachi Pakistan; ^3^ Department of Cardiology, Leonard M. Miller School of Medicine University of Miami Miami Florida USA; ^4^ Division of Cardiology Creighton University Omaha Nebraska USA; ^5^ Division of Cardiology East Carolina University Greenville North Carolina USA; ^6^ Division of Cardiology The University of Texas Health Sciences Center San Antonio Texas USA

**Keywords:** blood pressure, hyperlipidemia, hypertension, rosuvastatin, telmisartan

## Abstract

**Background:**

Hypertension often coexists with dyslipidemia, requiring combination therapy. Telmisartan, combined with amlodipine or rosuvastatin, targets these conditions. This meta‐analysis evaluates the efficacy and safety of these combinations in adults with hypertension and dyslipidemia.

**Methods:**

A systematic search was conducted in Cochrane Central, MEDLINE/PubMed, ClinicalTrials.gov, and ScienceDirect (as of June 2024) for randomized controlled trials (RCTs) comparing telmisartan plus amlodipine versus telmisartan plus rosuvastatin in adults (≥ 18 years) with hypertension and dyslipidemia. A random‐effects model was used with RevMan 5.4.1. The risk of bias and heterogeneity were assessed with the Cochrane Risk of Bias Tool and the *I*² statistic.

**Results:**

Three RCTs involving 320 participants were included. At 4 weeks, telmisartan + amlodipine yielded greater sSBP (sitting Systolic Blood Pressure) reduction compared to telmisartan + rosuvastatin (MD = −10.93 mmHg; 95% CI: −19.02 to −2.83; *p* = 0.008; *I*² = 70%). sDBP (sitting Diastolic Blood Pressure) reductions were greater in the amlodipine group at 8 weeks (MD = −8.59 mmHg; 95% CI: −13.35 to −3.82; *p* = 0.0004; *I*² = 58%). Conversely, LDL‐C reduction was favored by telmisartan + rosuvastatin, with significant effects observed at both 4 weeks (MD = 85.98 mg/dL) and 8 weeks (MD = 79.75 mg/dL). TEAE incidence did not differ significantly (RR = 1.23; 95% CI: 0.75–2.04; *p* = 0.41; *I*² = 0%).

**Conclusion:**

Telmisartan + amlodipine demonstrates superior antihypertensive efficacy, while telmisartan + rosuvastatin more effectively lowers LDL‐C. Safety profiles are comparable. Findings support the selection of a regimen based on individualized therapeutic goals.

**Abbreviations**
LDL‐C: low‐density lipoprotein cholesterol
MD: mean difference
RCTs: randomized controlled trials
RR: risk ratios
sDBP: sitting diastolic blood pressure
sSBP: sitting systolic blood pressure
TEAEs: treatment‐emergent adverse events

## 1 Introduction

Hypertension remains a significant global public health challenge, given its pivotal role as a precursor to cardiovascular diseases such as heart attack, stroke, and kidney failure. In light of this, combination therapies have gained significant attention for their enhanced efficacy in managing hypertension and associated risks [1]. Notably, the combination of Telmisartan with Amlodipine and Telmisartan with Rosuvastatin offers a dual approach that targets hypertension and the common cardiovascular comorbidity of dyslipidemia [2].

Telmisartan, an angiotensin II receptor blocker (ARB), works by inhibiting the effects of angiotensin II, resulting in vasodilation and a reduction in blood pressure [3]. Amlodipine, a calcium channel blocker (CCB), complements this by relaxing the blood vessels and reducing the heart's workload, further enhancing blood pressure control [4]. In contrast, rosuvastatin, a potent statin, lowers low‐density lipoprotein cholesterol (LDL‐C) levels by inhibiting HMG‐CoA reductase, thus addressing the lipid abnormalities often seen in hypertensive patients [2, 5].

While guidelines endorse combination therapy (ACC/AHA 2019, ESC/ESH 2023), there remains a scarcity of direct comparative evidence between antihypertensive and lipid‐lowering regimens. This meta‐analysis endeavors to address this gap by presenting quantitative comparisons and safety profiles of these combination therapies, assessing their influence on blood pressure regulation, cardiovascular health, and overall patient outcomes. By aggregating data from various studies, this review seeks to provide a comprehensive understanding of how these combinations can optimize treatment strategies, especially for high‐risk hypertensive patients. This may ultimately facilitate more personalized and efficacious therapeutic interventions.

## 2 Materials and Methods

The Preferred Reporting Items for Systematic Reviews and Meta‐Analyses (PRISMA) criteria, a set of guidelines for reporting systematic reviews and meta‐analyses, and the Assessment of Multiple Systematic Reviews (AMSTAR) 2, were both followed in conducting this systematic review and meta‐analysis [6, 7]. Also, this meta‐analysis was registered on PROSPERO (CRD42024563162). These criteria are designed to ensure transparency and completeness in reporting systematic reviews, which is crucial for the reliability and reproducibility of research findings. In our study, adherence to the PRISMA criteria ensures that our review is comprehensive, transparent, and methodologically sound, thereby enhancing the trustworthiness of our findings.

### 2.1 Data Sources and Search Strategy

We extensively searched the MEDLINE database via PubMed, the Cochrane Library, and ScienceDirect until June 20, 2024. Online databases, such as www.clinicaltrials.gov, were also searched to identify gray literature. The search strategy used MeSH terms and keywords related to telmisartan, amlodipine, rosuvastatin, hypertension, and dyslipidemia. The specific MeSH terms and keywords used in the search included “telmisartan,” “amlodipine,” “rosuvastatin,” “hypertension,” “dyslipidemia,” and “RCT.” Boolean operators, such as “AND,” “OR,” and “NOT,” were used to refine the search and ensure comprehensive coverage. We used a combination of these terms and Boolean operators to provide an extensive search. No language restrictions were applied during the search to ensure the broadest possible inclusion of studies. Two researchers independently carried out each search procedure. The precise search technique, including the specific terms and Boolean operators employed, is shown in Supporting Information S1: Table 1A.

### 2.2 Study Selection

After extracting every article from the databases, we moved it to EndNote X9 (ClarivateTM) to eliminate duplicates. Independently, two investigators in the group reviewed the titles and abstracts of the potentially relevant publications before reading the complete texts. Every step of the screening procedure was recorded. Inclusion criteria for randomized controlled trials (RCTs) were: (1) Participants who had hypertension and dyslipidemia; (2) Subjects were 18 years of age or older; (3) The intervention consisted of telmisartan plus amlodipine with a telmisartan plus rosuvastatin control group for comparison; and (4) Studies reported at least one of the outcomes of interest: Change in (sSBP) sitting systolic blood pressure, (sDBP) sitting diastolic blood pressures (sDBP), and LDL‐C at 4 and 8 weeks and treatment‐emergent adverse events (TEAES). Exclusion criteria included studies that did not meet the inclusion criteria, non‐RCTs, and studies that did not provide sufficient data for extraction. The primary studies were ultimately identified from four analyzed RCTs. There were no language‐based exclusions in the selection of randomized control studies.

### 2.3 Data Extraction

Two researchers independently extracted the data from the included articles using a self‐created information extraction form. Discrepancies were resolved through discussion or consultation with a third researcher. The study name and year of publication, study design, mean age of patients in each group, number of patients in each group, BMI, lipid levels, and all outcomes of interest were collected from each study. The primary outcome was the change in sitting SBP between the telmisartan plus amlodipine and telmisartan plus rosuvastatin groups from baseline at the fourth and eighth weeks. Secondary outcomes included changes in sDBP at the fourth and eighth weeks, LDL‐C at the fourth and eighth weeks, and TEAES.

### 2.4 Risk of Bias Assessment

We evaluated the risk of bias in the included RCTs using the Risk of Bias 2 tool (RoB 2) as advised by the Cochrane Collaboration. The RoB 2 tool evaluates five domains: the randomization process, deviation from intended intervention, missing outcome data, measurement of the outcome, and selection of the reported result. Additionally, outcome assessment and selection bias within published results were also taken into account when evaluating the research. Each study was thoroughly examined before being assigned a bias risk rating of “low risk,” “unclear risk,” or “high risk.” Two independent reviewers conducted the RoB assessment and resolved any disagreements. In dispute, a third reviewer was consulted to reach a consensus.

### 2.5 Statistical Analysis

We used ReviewManager (RevMan Version 5.4.1) (Cochrane et al. UK) for statistical analysis. Risk ratios (RR) were used to portray dichotomous outcomes, while the mean difference (MD) and standard deviation (SD) were used to present continuous outcomes. A random effects model was used. The 95% confidence interval (CI) was calculated for each effect size estimate. The heterogeneity among the included studies was presented using Higgins's *I* ^2^ measure, where a value of I2 less than 50% was considered mild heterogeneity, a value between 50% and 75% was regarded as moderate heterogeneity, and a value over 75% was considered severe heterogeneity [8]. The *p* less than 0.05 or equivalent was considered statistically significant. Beyond *I*², leave‐one‐out sensitivity analyses were conducted to test robustness; subgroup analyses by dose or baseline LDL were not possible due to limited trials.

## 3 Results

### 3.1 Study Selection

**Figure 1:**
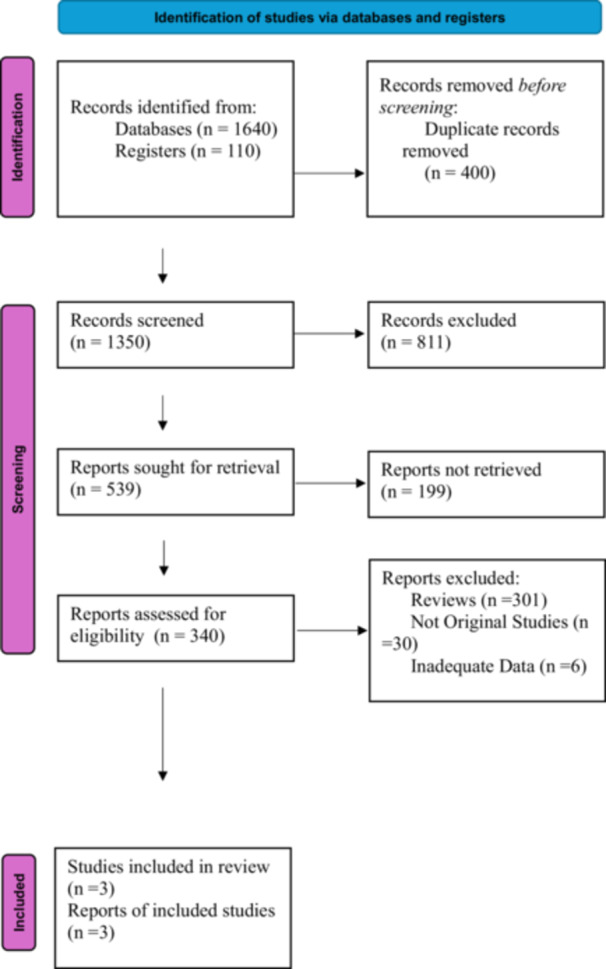
Prisma flow chart. After applying the inclusion and exclusion criteria, four studies were deemed eligible. Of these four trials, one was excluded due to the absence of extractable outcome data. Consequently, three randomized controlled trials (RCTs) with a combined total of 320 participants were included [5, 9, 10].

After applying the inclusion and exclusion criteria, four studies were deemed eligible. Of these four trials, one was excluded due to the absence of extractable outcome data. Consequently, three RCTs with a combined total of 320 participants were included [5, 9, 10]. The dosage regimens and treatment durations were analyzed across the trials and found to be consistent. Comprehensive characteristics are detailed in Tables 1 and 2. The process of study selection is depicted in Figure 1. Tables 1 and 2 provide a summary of the comprehensive characteristics of the studies and participants that were included.

**Table 1:** Characteristics of included studies.

Study characteristics						
Author name	Year	NCT	Population	Participants	Intervention	Control
Soon Jun Hong	2019	3088254	Hypertension plus dyslipidemia	97	Telmisartan/amlodipine	Telmisartan/rosuvastatin
Xuan Jin	2020	3067688	Hypertension plus dyslipidemia	131	Telmisartan/amlodipine	Telmisartan/rosuvastatin
Tae‐Seok Kim	2019	3566316	Hypertensive plus hyperlipidemia	92	Telmisartan/amlodipine	Telmisartan/rosuvastatin

**Table 2:** Baseline characteristics of patients included in the meta‐analysis.

Baseline characteristics																												
Author name	Year	NCT number	Participants		Sex				Age				BMI (kg/m²)				LDL‐C (mg/dL)				sitSBP (mmHg)				sitDBP (mmHg)			
			TA	TR	TA		TR		TA		TR		TA		TR		TA		TR		TA		TR		TA		TR	
					Male	Female	Male	Female	Mean	SD	Mean	SD	Mean	SD	Mean	SD	Mean	SD	Mean	SD	Mean	SD	Mean	SD	Mean	SD	Mean	SD
Soon Jun Hong	2019	NCT03088254	49	48	31	18	34	14	66.63	10.22	65.88	9.13	26.5	2.92	27.6	3.4	153.12	35.18	158.92	30.62	144.29	11.09	147.08	13.69	77.84	12.78	83.33	12.89
Xuan Jin	2020	NCT03067688	66	65	48	18	46	19	66.09	10.43	64.89	9.11	26.6	2.8	26.6	3.2	153.41	31.3	153.88	36.73	155	12.09	154.42	12.86	89.49	9.99	88.54	11.17
Tae‐Seok Kim	2019	NCT03566316	43	49	27	16	37	12	66.4	11	63.4	9.7	NR	NR	NR	NR	155.7	23.1	160	32	154.8	10.6	154.8	10.6	90.3	8.1	91	9.8

## 4 Changes in Outcome From Baseline

**Figure 2:**
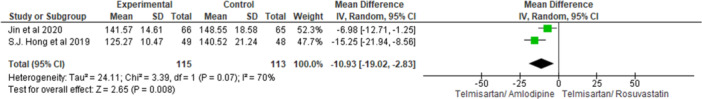
Change in sitting systolic blood pressure [sSBP] at 4th week.

**Figure 3:**
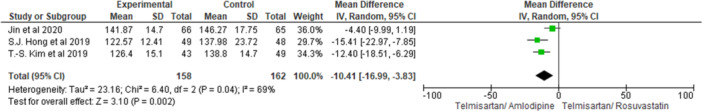
Change in sitting systolic blood pressure [sSBP] at 8th week.

Change in SSBP:

1. Change in sSBP at 4th Week:
  sSBP serves as a crucial indicator for assessing hypertension, and it was evaluated to compare the efficacy of both treatment groups. Two studies documented this outcome: Jin et al. and Hong et al. [5, 10]. Patients who received treatment with Telmisartan and Amlodipine demonstrated a significant reduction when compared to patients treated with Telmisartan and Rosuvastatin after 4 weeks (MD: −10.93; 95% CI: −19.02 to −2.83; *p*: 0.008; *I*² = 70%). Moderate heterogeneity was observed (*I*² = 70%); however, this was not statistically significant (*p* = 0.07) (Figure 2).
2. Change in sSBP at 8th Week:
  Three studies, namely Jin et al., Hong et al., and Kim et al., [5, 9, 10] reported a significant decline in sSBP after 8 weeks among patients treated with Telmisartan and amlodipine when compared to those administered Telmisartan and Rosuvastatin (MD: −10.41; 95% CI: −16.99 to −3.83; *p* = 0.002). A moderate level of statistically significant heterogeneity was observed across the three studies (*I*² = 69%; *p* = 0.04), indicating slight inconsistency in the findings. The leave‐one‐out sensitivity analysis affirmed the robustness of this conclusion. Specifically, the removal of individual studies did not significantly alter the pooled effect estimate, suggesting that no single study had an outsized influence on the overall results (Figure 3).

**Figure 4:**
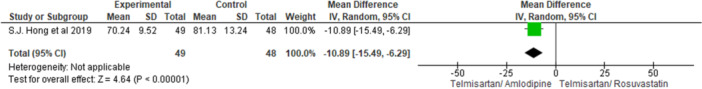
Change in sitting diastolic blood pressure [sDBP] at 4th week.

**Figure 5:**
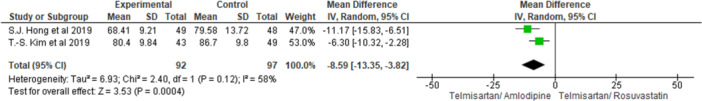
Change in sitting diastolic blood pressure [sDBP] at 8th Week.

Change in DBP:

1. Change in sDBP at 4th Week:
  To compare the efficacy of the drug groups Telmisartan and Amlodipine with those receiving Telmisartan and Rosuvastatin, sDBP and sDBP, critical parameters for assessing hypertension, were measured. Only one study provided this outcome, conducted by Hong et al. [5], which demonstrated a significant reduction in sitting DBP after 8 weeks of treatment with Telmisartan and Amlodipine when compared to the cohort receiving Telmisartan and Rosuvastatin (MD: −10.89; 95% CI: −15.49 to −6.29; *p* value: < 0.01) (Figure 4).
2. Change in sDBP at 8th Week:
  Two studies, conducted by Hong et al. and Kim et al. [5, 9] reported a significant reduction in sDBP after 8 weeks in patients administered Telmisartan and Amlodipine compared to those receiving Telmisartan and Rosuvastatin (MD: −8.59; 95% CI: −13.35 to −3.82; *p*: 0.0004). The findings across the two studies were notably consistent (*I*² = 58%; *p* = 0.12) (Figure 5).

**Figure 6:**
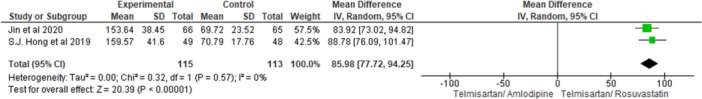
Change in low‐density lipoprotein cholesterol (LDL‐C) at 4th Week.

**Figure 7:**
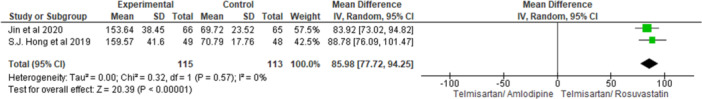
Change in low‐density lipoprotein cholesterol (LDL‐C) at 8th Week.

Change in LDL‐C Levels:

1. Change in LDL‐C Levels in 4th Week:
  LDL‐C levels were used as primary indicators of dyslipidemia to evaluate the efficacy of two distinct treatment regimens. Two studies reported by Jin et al. and Hong et al. demonstrated a significant reduction in LDL‐C levels after 4 weeks in the cohort receiving Telmisartan and Rosuvastatin, when compared to the group treated with Telmisartan and Amlodipine (MD: 85.98; 95% CI: 77.72 to 94.25; *p*: < 0.01) [5, 10]. The heterogeneity value was assessed as zero, indicating remarkable consistency across both studies (Figure 6).
2. Change in LDL‐C LEVELS at 8th Week:
  Three studies conducted by Jin et al., Hong et al., and Kim et al. reported a decrease in LDL‐C levels following 8 weeks of treatment for the group administered Telmisartan and Rosuvastatin, in contrast to the group receiving Telmisartan and Amlodipine (MD: 79.75 mg/dL; 95% CI: 65.15 to 94.35; *p* < 0.01; *I*² = 82%) [5, 9, 10]. A leave‐one‐out sensitivity analysis revealed that, despite the high heterogeneity, excluding any individual study did not significantly alter the direction or statistical significance of the effect size (Figure 7). Heterogeneity may also originate from dose titration protocols, variability in LDL assays, and discrepancies in blood pressure measurement techniques (office vs. automated).

## 5 TEAES

**Figure 8:**
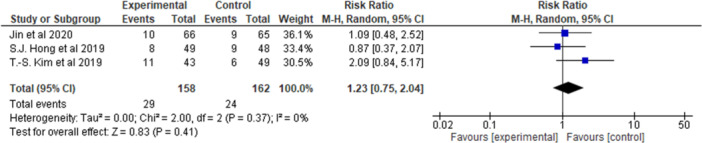
Treatment emergent adverse events.

In the context of the three eligible randomized trials (*n* = 320), a total of 29 out of 158 patients (18.4%) in the telmisartan + amlodipine group and 24 out of 162 patients (14.8%) in the telmisartan + rosuvastatin group experienced TEAES [5, 9, 10]. The meta‐analysis, using a random‐effects model, yielded a pooled relative risk (RR) of 1.23 (95% CI, 0.75–2.04; *p* = 0.41), indicating no statistically significant difference in overall TEAE risk between the two fixed‐dose combinations. The estimates at the study level remained consistent (RR range 0.87–2.09), and the heterogeneity was found to be negligible (*I*² = 0%). These findings suggest that, over the evaluated treatment duration of 4 to 8 weeks, telmisartan/amlodipine exhibits a safety profile comparable to that of telmisartan/rosuvastatin in hypertensive patients, with no definitive evidence of an increase in TEAEs (Figure 8).

## 6 Risk of Bias Assessment

Overall, the observed bias was minimal. All three RCTs demonstrated a low risk of bias concerning randomization [5, 9, 10], blinding (including participants, personnel, and assessors), and attrition. It is noted that only Hong et al. [5] presented unclear allocation concealment and instances of selective reporting, whereas Kim et al. [9] reported an unclear designation of “other bias”; no study registered a high‐risk classification in any domain. With over 90% of the assessments rated as low risk, the methodological integrity of the evidence base appears robust and unlikely to introduce bias into our pooled estimates. This is demonstrated in (Supporting Information S1: Figures 1 and 2 and Supporting Information S1: Table 2).

## 7 Discussion

This review assesses the efficacy and safety of two fixed‐dose combinations: Telmisartan with Amlodipine versus Telmisartan with Rosuvastatin, focusing on SBP, DBP, LDL‐C, and TEAEs during 4‐ and 8‐week treatments. Telmisartan with Amlodipine shows superior antihypertensive effects, with significant SBP reductions at 4 and 8 weeks, confirmed by sensitivity analyses showing robustness despite some heterogeneity. These findings align with their pharmacodynamics: telmisartan, an ARB, and amlodipine, a CCB, act synergistically to promote vasodilation, reduce resistance, and preserve renal perfusion without reflex tachycardia. DBP reductions also favored this combination, though limited studies restrict external validity.

The observed effect aligns with existing pharmacodynamic data; specifically, telmisartan, an ARB, and amlodipine, a dihydropyridine CCB, operate synergistically to promote vasodilation, reduce peripheral resistance, and sustain renal perfusion without inducing reflex tachycardia. This is particularly advantageous for patients with a high cardiovascular risk or resistant hypertension [11]. In contrast, telmisartan and rosuvastatin demonstrated superior lipid‐lowering efficacy, significantly diminishing LDL‐C levels. Additionally, telmisartan may exert further metabolic effects through partial activation of peroxisome proliferator‐activated receptor gamma (PPAR‐γ), which could enhance lipid modulation [12].

TEAEs occurred in 18.4% of patients with telmisartan + Amlodipine and 14.8% with telmisartan + Rosuvastatin across three trials. The pooled risk ratio showed no significant difference in TEAE risk between treatments. Study‐level estimates were consistent with minimal heterogeneity, supporting the safety comparison. Both combos are generally well‐tolerated short‐term, allowing clinicians to focus on efficacy (blood pressure vs. lipid control) based on patient goals without worries about side effects.

The findings presented in this study support the recommendations established by prominent clinical guidelines, including those issued by the American College of Cardiology/American Heart Association (ACC/AHA), and the European Society of Cardiology/European Society of Hypertension (ESC/ESH), which advocate for individualized combination therapy in patients exhibiting multiple cardiometabolic risk factors [13]. Furthermore, these findings strengthen the guideline‐recommended practices of individualized combination therapy for such patients. However, in contrast to the existing guidelines, this meta‐analysis provides quantitative comparative data aimed at guiding the selection of fixed‐dose combinations. For example, the combination of Telmisartan and amlodipine may be considered more advantageous for newly diagnosed hypertensive patients who require prompt blood pressure regulation or for those with normolipidemia. Conversely, the combination of Telmisartan and Rosuvastatin may be more appropriate for patients with a history of acute coronary syndromes (ACS) or those presenting with atherosclerotic cardiovascular disease, especially in cases where lowering LDL‐C is of utmost importance [12, 14].

Multiple analyses have indicated a moderate to high level of heterogeneity, particularly concerning SBP and LDL‐C outcomes observed at the 8‐week interval. This variation is likely attributable to several factors, including discrepancies in baseline characteristics among study participants, fluctuations in dosing protocols, and inconsistencies in measurement methodologies utilized across the trials. Furthermore, it is noteworthy that only a single study provided diastolic blood pressure data at the 4‐week interval, which constrains the robustness of conclusions drawn for this parameter. The limited scope of reported outcomes reduces the generalizability of the findings and restricts a comprehensive understanding of short‐term treatment effects. These considerations highlight the importance of interpreting the results within the context of methodological heterogeneity and data availability.

Clinical application: TA (telmisartan + amlodipine) may be preferred for patients with uncontrolled hypertension in the absence of dyslipidemia, whereas TR (telmisartan + rosuvastatin) is considered optimal for individuals at elevated risk of ASCVD. Further research should focus on assessing the reduction of cardiovascular events and medication adherence associated with fixed‐dose regimens over a period exceeding 1 year.

Future research: Future research should go beyond short‐term effects to assess long‐term cardiovascular event reduction, blood pressure and lipid control durability, and real‐world adherence to fixed‐dose regimens. Large trials with over 1‐year follow‐up are needed to determine if these combinations offer different benefits for outcomes like myocardial infarction, stroke, heart failure hospitalization, and mortality. Comparing these with emerging triple therapies (e.g., telmisartan–amlodipine–rosuvastatin) could help identify the best strategies for high‐risk patients.

## 8 Limitation

This study presents several limitations that warrant consideration when interpreting the findings. First, the limited number of RCTs included restricts the generalizability of the results and heightens the potential for publication bias. The reliance on published aggregate data rather than individual patient data limits our ability to conduct detailed subgroup analyses or adjust for confounding factors. Furthermore, the relatively brief follow‐up period of up to 8 weeks limits our ability to evaluate long‐term efficacy and safety outcomes. Variability in baseline patient characteristics, along with differences in dosing regimens across the studies, may also contribute to the heterogeneity observed in the results.

Additionally, only a single study provided data on diastolic blood pressure at the 4‐week time point, which restricts the robustness of conclusions drawn regarding this outcome. Finally, the reporting of TEAES was based on relatively small sample sizes, which may have failed to capture rare yet clinically significant adverse effects adequately. These considerations highlight the necessity for cautious interpretation and refinement in future research. In conclusion, the limited follow‐up duration (a maximum of 8 weeks) constitutes a significant limitation, as longer‐term outcomes, including major cardiovascular events, adherence trends, and quality‐of‐life metrics, were not assessed.

## 9 Conclusion

This systematic review and meta‐analysis provide a comprehensive, evidence‐based comparison of two widely utilized antihypertensive and lipid‐lowering combinations. The combination of telmisartan and amlodipine exhibited superior blood pressure reduction, whereas the combination of telmisartan and rosuvastatin demonstrated greater efficacy in lowering LDL cholesterol levels. Importantly, both combinations displayed comparable short‐term safety profiles, thereby supporting their application in personalized cardiometabolic care. These short‐term findings suggest differential benefits of each regimen; confirmation in larger, long‐term RCTs with cardiovascular outcomes is required before broad clinical adoption.

## Author Contributions

Rabia Asim, Sivaram Neppala, and Tazheen Saleh Muhammad contributed to the analysis and composed the original manuscript. Sivaram Neppala, Harigopal Sandhyavenu, Binish Qureshi, and Himaja Dutt Chigurupati supervised the study, while the other authors contributed to the analysis, manuscript preparation, and the creation of figures.

## Ethics Statement

The authors have nothing to report.

## Conflicts of Interest

The authors declare no conflicts of interest.

## Supporting information


**Supplementary Table 1A:** Search Strategy. **Supplementary Table 2:** Risk of Bias Summary. **Supplementary Figure 1:** Risk of Bias Summary. **Supplementary Figure 2:** Risk of Bias Graph.

## Data Availability

The data that supports the findings of this study are available in the Supporting Information S1 Material of this article.
