# Supplementary material for: Dual‐Targeted Therapy in Cardiometabolic Risk: A Meta‐Analysis of Telmisartan‐Based Combinations for Hypertension and Dyslipidemia
Source: Clin Cardiol. 2025 Nov 26;48(12):e70211. doi: 10.1002/clc.70211 (PMC12647966; doi:10.1002/clc.70211)

**Supplementary Table 1A.** Search Strategy

| **Database** | **Exact Boolean strategy*** |
| --- | --- |
| **PubMed/MEDLINE** | ((“Telmisartan”[Mesh] OR telmisartan[tiab]) AND (“Amlodipine”[Mesh] OR amlodipine[tiab]) OR (“Telmisartan”[Mesh] OR telmisartan[tiab]) AND (“Rosuvastatin Calcium”[Mesh] OR rosuvastatin[tiab])) AND (“Hypertension”[Mesh] OR hypertension[tiab] OR “Dyslipidemias”[Mesh] OR dyslipidemia[tiab] OR hyperlipidemia[tiab]) AND (randomized controlled trial[pt] OR random*[tiab] OR placebo[tiab]) |
| **Cochrane Library (CENTRAL)** | (“telmisartan” AND “amlodipine”) OR (“telmisartan” AND “rosuvastatin”) AND (hypertension OR dyslipidemia OR hyperlipidemia) AND (randomized OR placebo) |
| **Google Scholar** | (“telmisartan amlodipine” OR “telmisartan rosuvastatin”) AND (hypertension OR dyslipidemia OR “blood pressure” OR lipids) AND (randomized OR placebo) |
| **ScienceDirect** | (“telmisartan amlodipine” OR “telmisartan rosuvastatin”) AND (hypertension OR dyslipidemia OR hyperlipidemia) AND (randomized OR placebo) |
| **ClinicalTrials.gov** | telmisartan AND amlodipine AND rosuvastatin AND (hypertension OR dyslipidemia) AND (randomized OR placebo) |

**Supplementary Table 2:** Risk of Bias Summary

|  | Bias | Risk of Bias | Author’s Judgement |
| --- | --- | --- | --- |
| **Soon Jun Hong et al. 2019** | **Random sequence**  **generation**  **(selection bias)** | Low risk | the study  Utilized o a web based online randomization system in a 1:1:1 ratio , ensuring a proper random sequence generation limiting selection bias |
|  | **Allocation concealment**  **(selection bias)** | Unclear risk | The use of an online randomization system suggests potential for adequate concealment, but the involvement of principal investigators in patient enrollment and assignment raises concerns about selection bias. |
|  | **Blinding of participants**  **and personnel**  **(performance bias)** | Low risk | Participants were blinded to the treatment allocation throughout the study |
|  | **Blinding of outcome**  **Assessment**  **(detection bias)** | Low risk | Double-blind design was employed, ensuring that both participants and assessors were blinded to treatment allocation. This minimizes bias in outcome assessment . |
|  | **Incomplete outcome**  **data (attrition bias)** | Low risk | The study had a low dropout rate (16/148) and no reported non-adherence to treatment. All patients were included in the safety analysis. These factors suggest a low risk of attrition bias |
|  | **Selective reporting**  **(Reporting bias)** | Unclear risk | The study do mention the primary and secondary outcomes of the study, but they do not indicate whether these outcomes were pre-registered in a trial registry or if they align with a specific protocol. |
|  | **Other bias** | Low risk | document does not provide a direct **overall assessment** of the risk of bias for the entire study. |
|  |  |  | the study  Utilized |
| **Xuan Jin MD et al.**  **2020** | **Random sequence**  **generation**  **(selection bias)** | Low Risk | an interactive web-based system in a 1:1:1 ratio ensuring a proper random sequence generation limiting selection bias. |
|  | **Allocation concealment**  **(selection bias)** | Low Risk | The lack of explicit details about how allocation was concealed from study personnel creates uncertainty about the effectiveness of this process. |
|  | **Blinding of participants**  **and personnel**  **(performance bias)** | low Risk | A double-dummy technique was used to maintain a double-blind study. |
|  | **Blinding of outcome**  **Assessment**  **(detection bias)** | Low Risk | Outcome assessors were blinded to treatment assignment, minimizing the risk of detection bias |
|  | **Incomplete outcome**  **data**  **(attrition bias)** | Low Risk | Some patients withdrew due to adverse events or non-compliance. However, the use of the Full Analysis Set (FAS) population and the availability of information about withdrawal reasons help to mitigate the potential impact of missing data on the results.. |
|  | **Selective reporting**  **(Reporting bias)** | Low Risk | . the study published all the major endpoints, including changes in **mean sitting systolic blood pressure (msSBP)**, **LDL-C levels**, and **other lipid profiles**. This suggests that the risk of **reporting bias** is likely **low** because all relevant and pre-specified outcomes appear to have been reported comprehensively |
|  | **Other bias** | Low Risk | No other bias reported |
|  |  |  |  |
|  | **Random sequence**  **generation**  **(selection bias)** | Low Risk | The study employed a **stratified block randomization method** using SAS software to generate the random sequence. This minimizes the risk of selection bias. |
| **Tae-Seok Kim et al. 2019** | **Allocation concealment**  **(selection bias)** | Low Risk | .  Allocation concealment was ensured by the use of **identical placebos** and the double-dummy technique. The random assignment code was disclosed only in cases of significant medical events. |
|  | **Blinding of participants**  **and personnel**  **(performance bias)** | Low risk | Allocation concealment was ensured by the use of **identical placebos** and the double-dummy technique. The random assignment code was disclosed only in cases of significant medical events.. |
|  | **Blinding of outcome**  **Assessment**  **(detection bias)** | Low Risk | Outcome assessors were also blinded, ensuring objective evaluation of results such as changes in blood pressure and lipid levels. |
|  | **Incomplete outcome**  **data (attrition bias)** | Low Risk | Some patients withdrew, primarily due to adverse events, but the analysis included the **Full Analysis Set (FAS)**, reducing the risk of attrition bias by including all available data.. |
|  | **Selective reporting**  **(Reporting bias)** | Low Risk | All pre-specified outcomes, such as blood pressure and lipid changes, were reported. There is no indication of selective reporting. |
|  | **Other bias** | Unclear Risk | The study was funded by a pharmaceutical company, which could introduce **sponsorship bias**, although the authors stated that the sponsor had no role in study design, data collection, or analysis. |

**Supplementary Figure 1:** Risk of Bias Summary


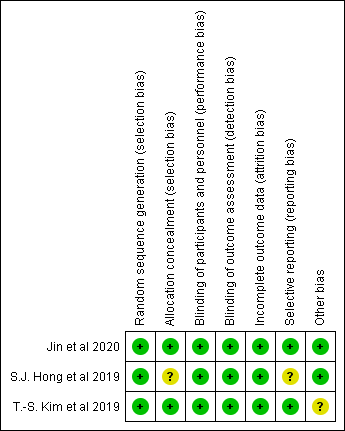


**Supplementary Figure 2:** Risk of Bias Graph


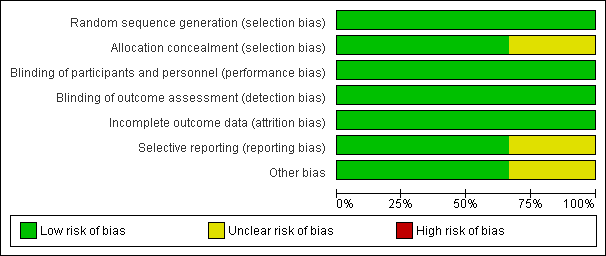

Supplement: Supplementary file 1 — Supplementary Table 1A: Search Strategy. Supplementary Table 2: Risk of Bias Summary. Supplementary Figure 1: Risk of Bias Summary. Supplementary Figure 2: Risk of Bias Graph. [file CLC-48-e70211-s001.docx]
